# Combined Exercise and Diet Induce Airway Hyperreactivity While Reducing Liver Steatosis in Mice with Diet-Induced Obesity

**DOI:** 10.3390/nu16132129

**Published:** 2024-07-03

**Authors:** Nora F. Marain, Anne-Charlotte Jonckheere, Ellen Dilissen, Jonathan Cremer, Tania Roskams, Marieke Colemont, Dominique M. Bullens, Lieven J. Dupont, Jeroen A. Vanoirbeek

**Affiliations:** 1 KU Leuven, Department of Chronic Diseases and Metabolism, Laboratory of Respiratory Diseases and Thoracic Surgery, 3000 Leuven, Belgium; 2 KU Leuven, Department of Microbiology, Immunology and Transplantation, Allergy and Clinical Immunology Research Group, 3000 Leuven, Belgiumdominique.bullens@kuleuven.be (D.M.B.); 3 KU Leuven, Department of Imaging & Pathology, Translational Cell & Tissue Research, 3000 Leuven, Belgium; 4 Clinical Division of Paediatrics, UZ Leuven, 3000 Leuven, Belgium; 5 Clinical Division of Respiratory Medicine, UZ Leuven, 3000 Leuven, Belgium; 6 KU Leuven, Department of Public Health and Primary Care, 3000 Leuven, Belgium

**Keywords:** obesity, exercise, fat intake, dietary

## Abstract

Background: Obesity is a multi-organ system disease, which is associated with, e.g., a higher prevalence of non-alcoholic fatty liver disease (NAFLD) and asthma. Little is known regarding the effect of obesity-related parameters (including liver integrity) and the respiratory phenotype after a combination of physical activity and diet. Methods: Thirty-two C57BL/6 mice were, after 27 weeks of a high fat diet (HFD), randomly assigned to two dietary interventions for three weeks: a HFD or a normal chow diet (NCD). In both dietary groups, half of the animals were subjected to a sub-maximal exercise protocol. Lung function, lung inflammation, liver histology, and metabolic profile were determined. Results: Mice with obesity did not show airway hyperreactivity after methacholine provocation. Sub-maximal exercise with diet (NCD/E) induced a significant reduction in forced expiratory volume in 0.1 s after methacholine provocation. NCD/E had significantly more neutrophils and inflammation (IFN-γ, TNF-α, IL-4, and IL-17F) in bronchoalveolar lavage compared to non-exercising mice on a HFD (HFD/NE). However, more epithelial injury (serum surfactant protein D and IL-33) was seen in HFD/NE. Additionally, hepatic steatosis and fibrosis were reduced by combined diet and sub-maximal exercise. Conclusions: Combining sub-maximal exercise with diet induced airway hyperreactivity and pulmonary inflammation, while body weight, hepatic steatosis, and fibrosis improved.

## 1. Introduction

Obesity is a metabolic disease caused by an imbalance between energy intake and expenditure [1,2,3,4,5]. Generally, there is an increased consumption of energy-dense foods and a decreased physical activity leading to abnormal or excessive fat accumulation [6]. This abnormal or excessive fat accumulation can result in impaired overall health, expressed as an increased risk to develop noncommunicable diseases such as cardiovascular diseases, diabetes, and some cancers. The worldwide prevalence of obesity has nearly tripled since 1975, with over 650 million adults suffering from obesity in 2016 [7]. The dramatically rising prevalence and increased risk for comorbidities emerges as a major public health problem [8]. Current recommendations in the treatment of obesity rely on diet supplemented by physical activity [9].

Obesity is a multi-organ system disease, affecting, amongst others, body weight, glucose metabolism, liver, and hepatocyte function [10]. Most commonly, obesity is associated with non-alcoholic fatty liver disease (NAFLD), which is characterized by an increased intrahepatic triglyceride content, defined as steatosis, in the absence of a history of significant alcohol use [11]. This steatosis can be accompanied by inflammation and fibrosis. Body weight reduction by at least three to five percent is required to reduce the steatosis [12]. Consequently, lifestyle modifications, whereby exercise and dietary restrictions are combined, are recommended as the most effective interventions [13]. Yet only a limited number of studies have been performed to evaluate the reduction in steatosis induced by physical exercise.

From epidemiological research, it is well known that obesity is a risk factor for both the incidence and prevalence of asthma [14]. Furthermore, obesity is associated with asthma severity and is a negative disease modifier for asthma, hence worsening asthma control [15]. Recent research has associated reduced lung function with the presence of NAFLD [16]. Different factors associated with obesity can contribute to the development of asthma-like symptoms or disease. For example, the mechanical functioning of the chest is affected by excess adipose tissue [17]. Moreover, adipose tissue can produce different inflammatory mediators, such as interleukin 6 (IL-6), that can affect the lungs [14].

Airway hyperresponsiveness (AHR) is a key feature of asthma [18]. Previous research has already shown that mice on a high-fat diet (HFD) can develop diet-induced obesity and AHR [19,20]. However, the underlying mechanisms are not fully understood.

Both exercise and diet, preferentially when combined, are majorly used as conservative measures in the treatment of obesity [9]. Therefore, we aimed to evaluate if intensive exercise can be used for the rapid treatment of obesity and what role diet plays in the effects on the respiratory system, the local and systemic inflammation, and the hepatological comorbidities of obesity.

The effect of exercise [6,21] and fat restriction [21] separately on obesity has already been investigated, but to our knowledge, no study has investigated the combined effect of exercise and diet, as currently suggested as treatment in humans, on the respiratory phenotype in mice. This allows us to study the effect of both triggers separately as well as combined on the liver and lungs. Here, we aim to investigate the effect of sub-maximal exercise with and without fat restriction on the respiratory system, the liver, and systemic inflammation in diet-induced obesity in mice.

## 2. Materials and Methods

### 2.1. Animals

Male C57BL/6Jax mice (n = 32) were obtained from Jackson Laboratory (Bar Harbor, ME, USA). All mice were housed per experimental group in individually ventilated cages in a conventional animal facility with 12 h dark/light cycles and had access to water and food ad libitum, and a handful of paper wool nesting material was provided. Room temperature was maintained at 22–24 °C and relative humidity at 50–60%. Approval for this study was given by the local Ethical Committee for Animal Experiments of KU Leuven, Leuven, Belgium (P065/2017). More detailed protocols can be found in Appendix A.

### 2.2. Experimental Protocol

**Figure A1:**
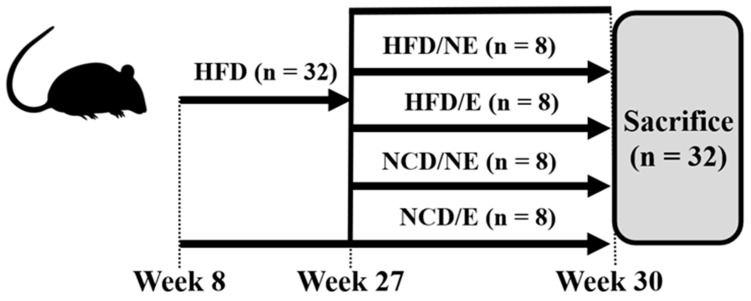
Experimental study design. HFD = high-fat diet, NCD = normal chow diet, NE = no exercise, E = exercise.

From 8 weeks of age, all mice received a high-fat diet (HFD) containing 60% fat (D12492, Research Diets, New Brunswick, NJ, USA). At 27 weeks of age, the animals were divided into four groups (Figure A1): (1) HFD group + no exercise (n = 8): continuation of a HFD (HFD/NE); (2) HFD + exercise group (n = 8): continuation of a HFD with an exercise protocol [22] (HFD/E); (3) NCD group + no exercise (n = 8): switch from a HFD to a normal chow diet (NCD) for the remaining 3 weeks of resting (NCD/NE); (4) NCD + running group (n = 8): switch from a HFD to a NCD for the remaining 3 weeks of the protocol with an exercise protocol (NCD/E).

The previously optimized sub-maximal exercise protocol was performed as described earlier [22]. On Day 1 and 2, mice acclimatized to the treadmill by first running 5 min without movement of the treadmill itself, immediately followed by 10 min at 6 m per min. On the third day, the mice performed an endurance test to determine their individual maximal running capacity. This test started with 5 min running at 3 m per min whereafter the speed was increased with 1 m per min until exhaustion. This was defined as failure to reach the end of the treadmill after mechanical stimulation. From Day 6, mice ran for 5 consecutive days, 30 min per day, for 3 consecutive weeks. Every day, the starting speed of the treadmill was set to 6 m per min for 5 min followed by a 1 m per min increase until 70% (Week 1), 75% (Week 2), and 80% (Week 3) of their maximal running speed. This speed was maintained until the mice ran 30 min. This exercise protocol was previously optimized to induce EIB in predisposed animals, without inducing AHR or inflammation in controls.

### 2.3. Lung Function Assessment

Lung function measurements were performed as described earlier [23]. Forced oscillation technique (FOT) maneuvers and forced expiratory measurements were performed using the flexiVent FX system (SCIREQ, Montreal, QC, Canada, v7.6 software), which was equipped with a FX2 module and a NPFE extension. A small-particle-size Aeroneb Lab nebulizer (2.5–4 µm, Aerogen, Galway, Ireland) was integrated in the inspiratory arm of the Y-tubing generating aerosol challenges at a 50% duty cycle for 5 s. Twenty-four hours after the last running or rest session, mice were anesthetized with pentobarbital (120 mg/kg body weight, Dolethal) and tracheotomized. After anesthesia, an 18 G metal cannula with a typical resistance of 0.3 cmH_2_O·s/mL was inserted. Mice were ventilated quasi-sinusoidally with a tidal volume of 10 mL/kg at a frequency of 150 breaths/min.

At the start of an experiment, two consecutive deep inflations were applied to maximally inflate the lungs to a pressure of 30 cm H_2_O to open the closed area and standardize the lung volume. The lungs were equilibrated at that pressure for 3 s. The gas compression-corrected volume is read as inspiratory capacity (IC). An automated sequence of five consecutive, closely spaced FOT measurements using the Quick Prime-3 (QP3), a 3 s long forced oscillation perturbation, was initiated. This sequence was followed by a negative pressure forced expiration (NPFE) maneuver to obtain forced expiratory parameters (forced expiratory volume at 0.1 s (FEV0.1), forced vital capacity (FVC), peak expiratory flows (PEF), and FEV0.1/FVC (referred to as the Tiffeneau–Pinelli index). This was performed by inflating the lungs to a pressure of 30 cm H_2_O over 1.2 s and then rapidly exposing the airways to a negative pressure of −55 cm H_2_O. Next, the same automated sequence was used for measuring airway hyperresponsiveness (AHR) and forced expiratory parameters in response to increasing concentrations of methacholine (0, 2.5, 5, 10, 20, 40 mg/mL). The sequence started immediately after the 5 s aerosol exposure.

Small airway resistance and reactance were calculated from the impedance data, as the difference in resistance between the lowest and highest frequencies tested in the Quick Prime-3 forced oscillation maneuver, as described earlier [24].

### 2.4. Blood Sampling

After lung function measurements, the mice received an overdose of pentobarbital, whereafter blood was sampled through cardiac puncture. To obtain plasma, 400 µL blood was added to precoated tubes with 8 µL of 0.5 mM EDTA, while the remaining blood was collected in an uncoated tube and used to obtain serum. All samples were centrifuged (14,000× *g*, 4 °C, 10 min), whereafter serum and plasma were stored at −80 °C until further analysis.

### 2.5. Broncho-Alveolar Lavage (BAL)

The lungs were three times lavaged with 0.7 mL sterile saline (0.9% NaCl), whereafter recovered fluid was pooled. BAL fluid was centrifuged (1000× *g*, 10 min), and resuspended cells were counted in a Bürker hemocytometer. A total of 250 µL of the cells was spun (300× *g*, 6 min, Cytospin 3, Shandon, TechGen, Zellik, Belgium) onto microscope slides, air-dried, and stained (Diff-Quick method, ThermoFisher Scientific, Waltham, MA, USA). In total, 200 cells per slide were counted to determine the number of macrophages, lymphocytes, eosinophils, and neutrophils.

### 2.6. Single Cell Suspension

During autopsy, the left lung lobe was collected and directly processed to a single cell suspension, as previously described [22], using digestion medium for 45 min at 37 °C. Thereafter, red blood cells were lysed with GibcoTM ammonium chloride potassium lysing buffer (ThermoFisher Scientific, Waltham, MA, USA). Finally, cells were counted in a Bürker hemocytometer and resuspended in PBS (10^7^ cells/mL). Subsequently, cells were stained for viability and labelled with anti-mouse fluorochrome-conjugated monoclonal antibodies to identify dendritic cell subpopulations. More detailed description can be found in Appendix A.

### 2.7. Surfactant Protein D Analysis

Surfactant protein D (SpD) concentration was measured in serum using the Mouse SP-D DuoSet ELISA (R&D Systems, Minneapolis, MN, USA) according to the manufacturer’s instructions. The detection limit was 62.5 µg/mL. Samples were undiluted.

### 2.8. DNA Methylation Assay

Genomic DNA was isolated from EDTA-coated blood pellet using the DNeasy Blood and Tissue kit (Qiagen, Hilden, Germany) according to the manufacturer’s instructions. The global percentage of 5-mC was determined using the MethylFlashTM Global DNA Methylation (5-mC) Elisa Easy Kit (Epigentek Group Inc., Farmingdale, NY, USA). In total, 100 ng of DNA was added to the high-DNA affinity strip wells, whereafter the 5-mC antibody was added. Absorbance was measured at 450 nm.

### 2.9. Cytokine Analysis

The cytokine and chemokine concentrations of interferon-γ (IFN-γ), IL-13, IL-17A, IL-4, IL-5, IL-6, IL-33, IL-17F, keratinocyte-derived chemokine (KC), and tumor necrosis factor-α (TNF-α) were measured in the BAL fluid, and the cytokine and hormone concentrations of IL-1β, IL-6, IL-17A, IL-17F, TNF-α, ghrelin, glucagon, insulin, leptin, and peptide YY (PYY) were measured in plasma using a U-plex Assay (Meso Scale Diagnostics, Rockville, MD, USA) according to the manufacturer’s instruction. The detection limits are available in Table A1.

### 2.10. Liver Histological Evaluation

During autopsy, the left liver lobe was collected, fixed in 4% formaldehyde, and embedded in paraffin for histological analysis.

Serial 10 µm-thick sections were stained with hematoxylin and eosin (H&E) and stained with Sirius Red for hepatic fibrosis. All liver samples were analyzed by an expert pathologist, blinded for intervention. Liver samples were scored for micro-vesicular steatosis, characterized by the presence of small vesicles of fat without displaying a nucleus, and macro-vesicular steatosis, characterized by the swelling of hepatocytes by a large fat droplet that displays a nucleus [25]. The intensity of steatosis was scored as 0 (<5%), 1 (5%–33%), 2 (34%–66%), or 3 (>66%). Fibrosis was scored as F0 (no fibrosis), F1 (Zone 3 perisinusoidal fibrosis (all) or portal fibrosis only), F2 (Zone 3 + periportal fibrosis), F3 (bridging fibrosis), or F4 (cirrhosis).

### 2.11. Lung Histological Evaluation

During autopsy, the lower right lung lobe was collected, fixed in 4% formaldehyde, and embedded in paraffin for histological evaluation. Serial 5 µm-thick sections were stained with periodic acid Schiff (PAS) staining. An experienced pathologist evaluated airway inflammation in a blinded manner.

### 2.12. Tight Junction mRNA Expression by qPCR

During autopsy, the upper right lung lobe was collected, immediately snap-frozen, and stored at −80 °C for RT-qPCR. The lungs were homogenized for RNA extraction and RNA was isolated using the Qiagen Mini RNeasy kit (Qiagen, Hilden, Germany) as described earlier [22]. In total, 1 µg RNA was converted into cDNA using the High-Capacity cDNA Reverse Transcription Kit (Applied Biosystems™, Foster City, CA, USA) in the presence of 1U RNAse inhibitor. cDNA was used to determine the gene expression of zona occludens-1 (TJP1), claudin-1 (CLDN1), claudin-3 (CLDN3), claudin-4 (CLDN4), claudin-18 (CLDN18), occludin (OCLN), and the reference genes α-actin (ACTB) and ribosomal protein L13a (RPL13A) via RT-qPCR as previously described [22].

### 2.13. Data Analysis

Data are represented as the individual mice and group mean or as mean with standard deviation (SD). Normality of the data was assessed using Shapiro–Wilk test. Hereafter, intergroup differences were evaluated using a one-way parametric ANOVA combined with a Bonferroni multiple comparison post hoc test or a non-parametric Kruskal–Wallis test with a Dunn’s multiple comparison post hoc. Combination effects were evaluated using a two-way ANOVA with a Bonferroni multiple comparison post hoc test. Data were evaluated using GraphPad Prism (9.3.0, GraphPad Software Inc., San Diego, CA, USA). A level of *p* < 0.05 was considered significant. No correction for multiple testing across parameters was performed since data were analyzed in an exploratory setting.

## 3. Results

### 3.1. Body Weight Evolution

**Figure 1:**
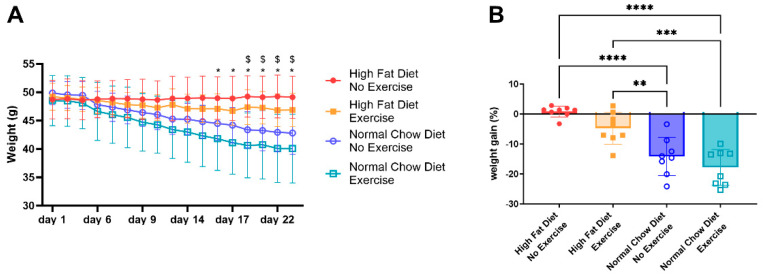
Body weight and weight loss. Body weight evolution (**A**). Data are represented as the mean ± SD. The levels of significance for the groups were * for NCD/E and $ for NCD/NE: * *p*, $ *p* < 0.05. Data were evaluated using two-way ANOVA with a Bonferroni multiple comparison post hoc test. Weight loss (**B**) represented as percentage of start weight. Data were evaluated with one-way ANOVA with a Bonferroni multiple comparison post hoc test and are represented as the mean ± SD with individual values. ** *p* < 0.01; *** *p* < 0.001; and **** *p* < 0.0001. n = 8 for all groups.

The evolution of the body weight is shown in Figure 1A. At the start of the protocol (Day 1), the average weight (±standard deviation) of all animals was 49.1 ± 3.1 g. The weight of the mice that were kept on the HFD without exercise (HFD/NE) and with exercise (HFD/E) remained stable over the entire protocol. The weight of the mice that received a normal chow diet from the start of the protocol declined fast. Exercising mice (NCD/E) weighed significantly less than HFD/NE mice from Day 16 onwards (*p* = 0.0458), whereas non-exercising mice (NCD/NE) became significantly lower in weight from Day 20 onwards (*p* = 0.0194), compared to HFD/NE mice. The total weight loss over the entire protocol is presented in Figure 1B. Exercise without a change of diet (HFD/E) did not result in significant weight loss compared to HFD/NE. Fat restriction both without (NCD/NE) and with exercise (NCD/E) resulted in significantly more weight loss compared to HFD/NE and to HFD/E.

### 3.2. Lung Function

#### 3.2.1. Baseline Lung Function

**Figure 2:**
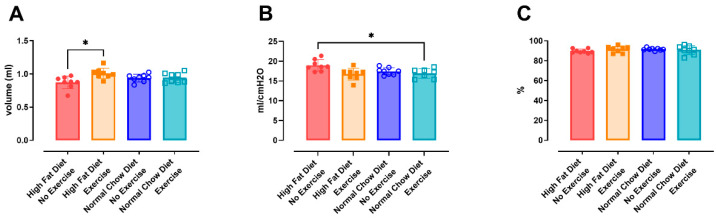
Baseline lung function. Baseline lung function and airway hyperreactivity to increasing concentrations of methacholine was assessed 24 h after the last running or rest session using the flexiVent system. Inspiratory capacity (**A**), tissue elasticity (**B**) and Tiffeneau–Pinelli index (**C**), calculated as FEV0.1/FVC, were evaluated using one-way ANOVA with a Bonferroni multiple comparison post hoc test. Data are represented as the mean ± SD with individual values. * *p* < 0.05. n = 7–8 per group.

At Week 30, after 3 weeks of exercise, a significantly higher baseline inspiratory capacity (*p* = 0.0267) in animals on a HFD with exercise (HFD/E) compared to HFD/NE was observed (Figure 2A). NCD/NE resulted in a significantly lower tissue elastance compared to HFD/NE (*p* = 0.0188) (Figure 2B). No other baseline lung function parameters (Rn, FVC, FEV0.1, FEV0.2, PEF) were different between groups (Figure A3A–E). The Tiffeneau–Pinelli index (TI) was calculated as the FEV0.1/FVC ratio as a measure of airway obstruction. None of the groups showed signs of airway obstruction (TI < 70%) and neither diet nor exercise could induce significant changes (Figure 2C).

**Figure A2:**
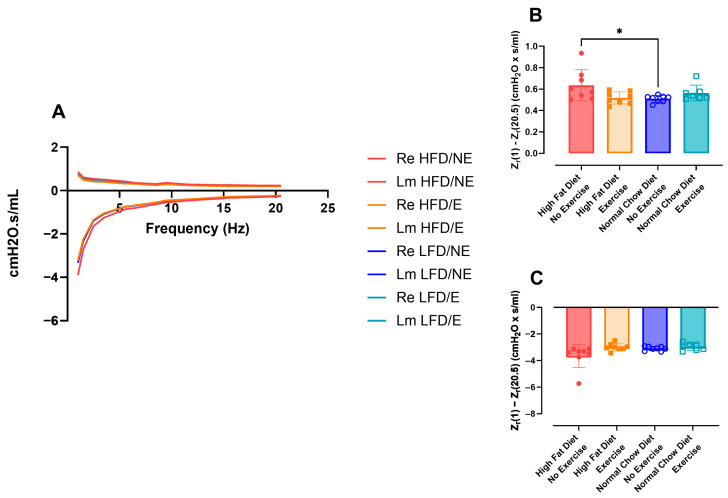
Small airway resistance and reactance. Respiratory impedance (**A**), measured with the forced oscillation technique over a range of 1 to 20.5 Hz showing the real (resistance) and imaginary (reactance) part of the impedance. Small airway resistance (**B**) and small airway reactance (**C**), calculated by subtracting the highest frequency impedance [Z (20.5 Hz)] from the lowest frequency impedance [Z (1 Hz)] measurement of the upper and lower curve, respectively. Data were evaluated using one-way ANOVA with a Bonferroni multiple comparison post hoc test. Data are represented as the mean with individual values. * *p* < 0.05. n = 7–8 per group.

The small airway resistance (Figure A2B), calculated with the impedance data (Figure A2A), of the NCD/NE group at baseline was significantly lower compared to the HFD/NE group (*p* = 0.0393), whereas the small airway reactance remained identical between groups (Figure A2C).

#### 3.2.2. Airway Hyperreactivity

**Figure A3:**
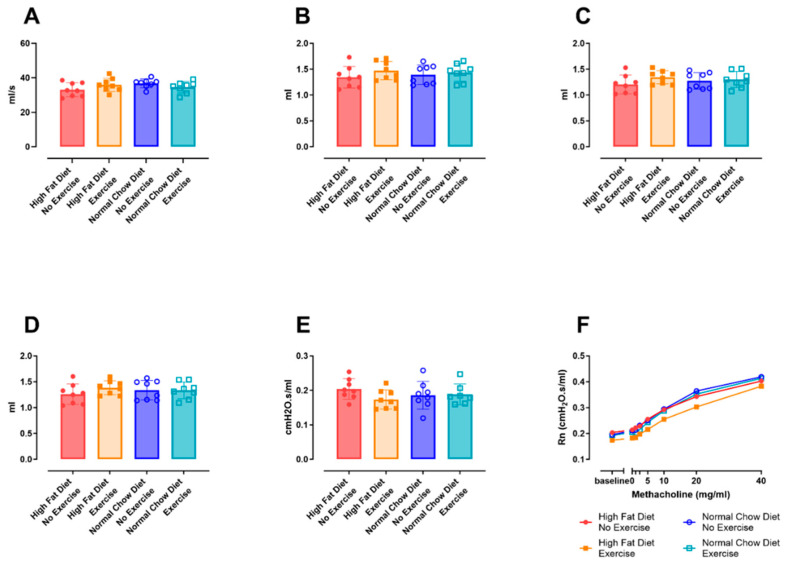
Baseline lung function and airway hyperreactivity. Lung function parameters and hyperreactivity were assessed 24 h after the last running or resting session using the flexiVent system. Baseline PEF (**A**), baseline forced vital capacity (FVC) (**B**), baseline FEV0.1 (**C**), baseline FEV0.2 (**D**), and baseline airway resistance (Rn) (**E**) are represented as the mean ± SD with individual values. Data were evaluated using one-way ANOVA with a Bonferroni multiple comparison post hoc test, n = 8 per group. The group average dose–response of Rn to increasing concentrations of methacholine (0–40 mg/mL) (**F**) was measured using the QP3 forced oscillation technique. Data were evaluated using two-way ANOVA with a Bonferroni multiple comparison post hoc test, n = 7–8 per group.

Airway hyperreactivity, measured with the forced oscillation perturbation QP3 and presented as airway resistance (Rn) to an increasing concentration of methacholine, was not different between groups (Figure A3F). The HFD/NE group did not show any signs of airway hyperreactivity.

**Figure 3:**
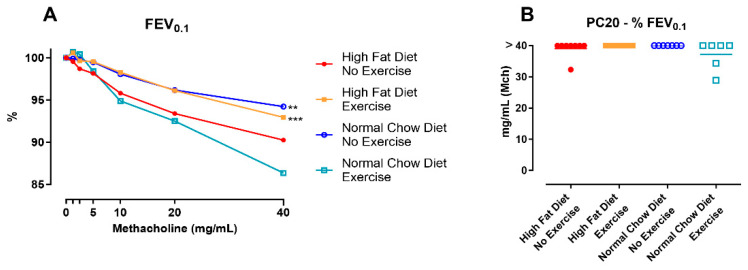
Airway hyperreactivity to increasing methacholine dose. The group-average dose–response FEV0.1 (%) to methacholine (0–40 mg/mL) (**A**) was acquired using the negative pressure forced expiration (NPFE) maneuver. Data were evaluated using two-way ANOVA with a Bonferroni multiple comparison post hoc test. The provocative concentration of methacholine needed to induce a 20% decrease of FEV0.1 compared to baseline FEV0.1 (PC20) (**B**) was compared using one-way ANOVA with a Bonferroni multiple comparison post hoc test. Data are represented as the mean with individual values. The levels of significance for the groups were * for comparison to NCD/E, ** *p* < 0.01; *** *p* < 0.001. n = 6–8 per group.

FEV0.1 (%) was significantly reduced at 40 mg/mL methacholine in the NCD/E group compared to the HFD/E (*p* = 0.0042) and NCD/NE (*p* = 0.0006) groups (Figure 3A). However, this was not reflected in the PC20, calculated based on a reduction of more than 20% in FEV0.1 (%), since the reduction in FEV0.1 did not exceed 10% in most animals (Figure 3B).

#### 3.2.3. Lung Inflammatory Response

**Figure 4:**
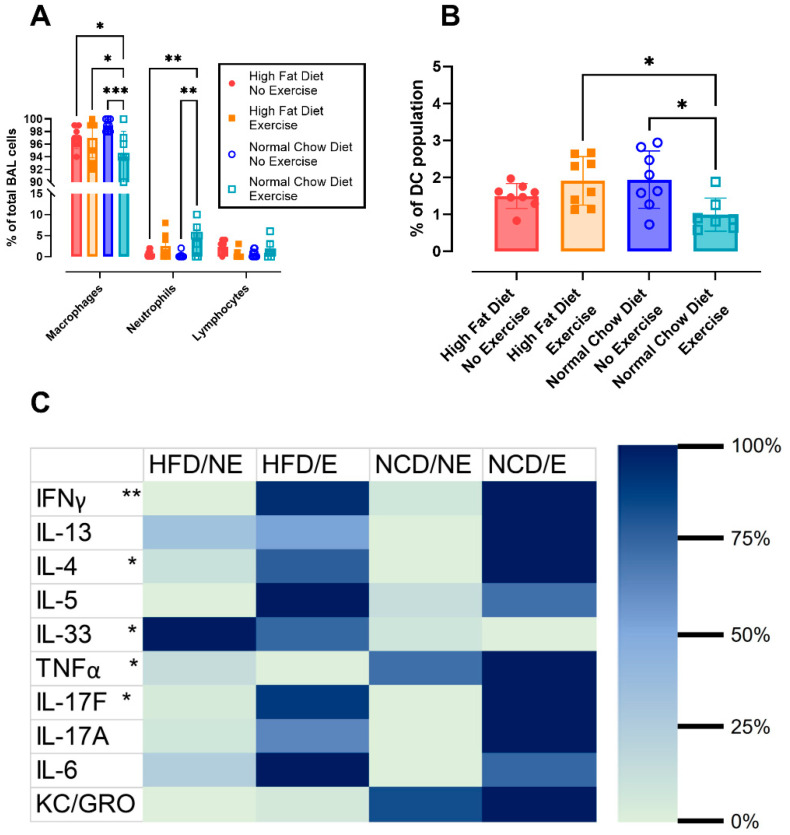
Lung inflammatory response. Percentual number of macrophages, neutrophils, and lymphocytes in bronchoalveolar lavage fluid (**A**). Data are represented as mean ± SD with individual values. Data were evaluated using two-way ANOVA with a Bonferroni multiple comparison post hoc test. CD45+ low auto-fluorescence MHCII+ CD11c+ SiglecH+ plasmacytoid DCs (pDCs, **B**) in lung tissue were assessed using flow cytometry. Data are represented as the mean ± SD with individual values. Data were evaluated using one-way ANOVA with a Bonferroni multiple comparison post hoc test. n = 7–8 per group. Concentration of cytokines in bronchoalveolar lavage (**C**). Data are represented as a heat map showing proportional differences between groups. Individual cytokines were compared using the Kruskal–Wallis test with Dunn’s post hoc testing. The levels of significance of NCD/E compared to HFD/NE were * *p* < 0.05, ** *p* < 0.01, *** *p* < 0.001. n = 8 for all groups.

In the bronchoalveolar lavage fluid, the differential cell count showed a significantly increased percentage of neutrophils in NCD/E mice compared to HFD/NE (*p* = 0.0083) and NCD/NE (*p* = 0.0020) (Figure 4A). There were no differences in the percentage of lymphocytes, and we did not find any eosinophils in the BAL.

**Figure A4:**
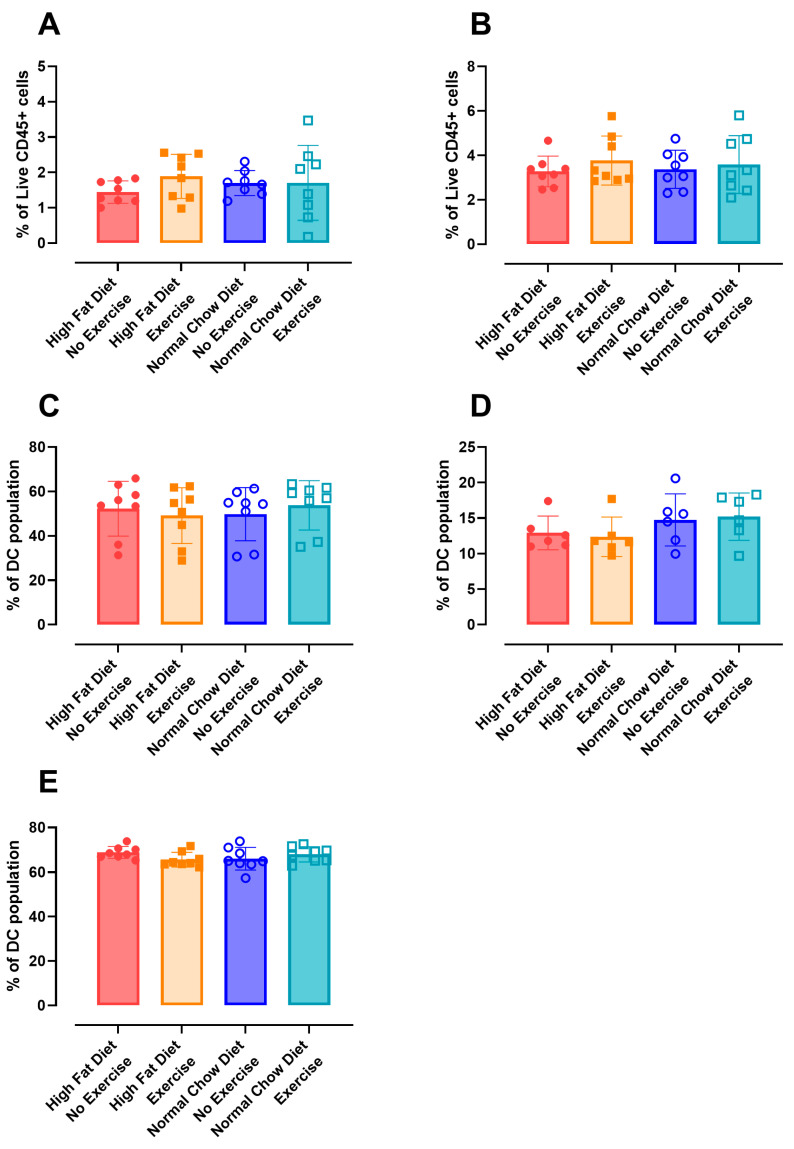
Cellular lung immune response. Macrophages and dendritic cell subpopulations were assessed using flow cytometry in lung tissue. Cells were analyzed as auto-fluorescent macrophages (**A**), CD45+ low auto-fluorescent MHCII+ CD11c+ DC (total DC population) (**B**), CD45+ low auto-fluorescent MHCII+ CD11c+ CD11b+ CD64+ monocyte derived DC (moDC) (**C**), CD45+ low auto-fluorescent MHCII+ CD11c+ CD11b- CD103+ conventional DC (CD103+ cDC) (**D**), and CD45+ low auto-fluorescent MHCII+ CD11c+ CD11b+ CD103- conventional DC (CD11b+ cDC) (**E**). Data are represented as the mean ± SD with individual values. Data were evaluated using the Kruskal–Wallis test with a Dunn’s multiple comparison post hoc test for moDC and one-way ANOVA with a Bonferroni multiple comparison post hoc test for all other parameters. n = 6–8 per group.

**Figure A5:**
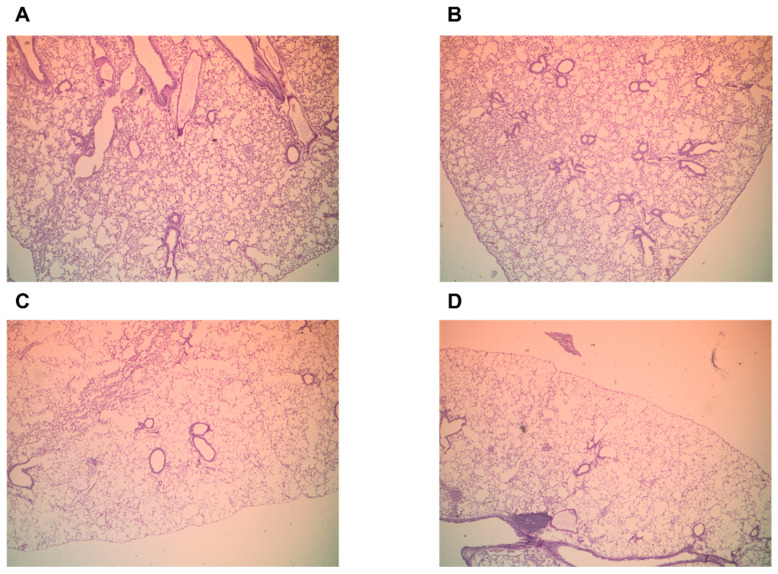
Histological evaluation of lung tissue. PAS-stained sections of lung tissue from HFD/NE (**A**), HFD/E (**B**), NCD/NE (**C**), and NCD/E (**D**). Magnification 4×.

The presence of macrophages and different subpopulations of dendritic cells (DC) in lung tissue was assessed using flow cytometry. No differences were seen in macrophages or total DCs (Figure A4A,B). Only plasmacytoid DCs were significantly lower in NCD/E compared to HFD/E (*p* = 0.0257) and NCD/NE (*p* = 0.0216) (Figure 4B). All other subpopulations (monocyte-derived DCs, CD11b-CD103+ DC, and CD11b+CD103- DC, Figure A4C–E) showed no differences between the groups. Additionally, on histological evaluation, no aberrant airway inflammation or epithelial damage could be seen (Figure A5A–C).

**Figure A6:**
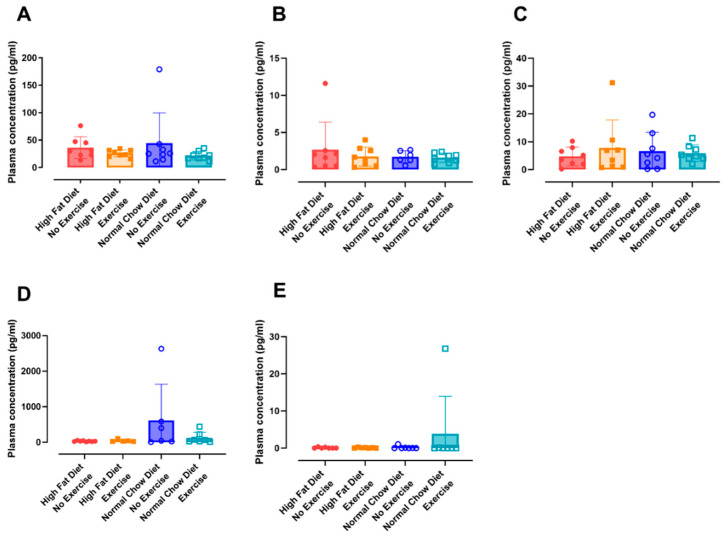
Systemic inflammatory response. Concentration of TNFα (**A**), IL-17A (**B**), IL-1β (**C**), IL-6 (**D**), and IL-17A/F (**E**) was measured in plasma using a U-plex Assay (Meso Scale Diagnostics). Data are represented as the mean ± SD with individual values. Data were evaluated using the Kruskal–Wallis test with a Dunn’s multiple comparison post hoc test. n = 7–8 per group.

The inflammatory response in the lungs was further evaluated by assessing the concentration of cytokines in the BAL fluid (Figure 4C). NCD/E induced significant increases in IFNγ (*p* = 0.0090), IL-4 (*p* = 0.0456), TNFα (*p* = 0.0115), and IL-17F (*p* = 0.0467) concentrations in the BAL compared to HFD/NE. IL-33 was significantly lower in NCD/E compared to HFD/NE (*p* = 0.0353). The concentration of IL-13, IL-5, IL-17A, IL-6, and KC/GRO did not differ between groups. Additionally, the inflammatory response was assessed in plasma to evaluate systemic inflammation. Yet, none of the investigated inflammatory parameters (TNFα, IL-1β, IL-6, IL-17A, and IL-17A/F) differed in plasma concentration between groups (Figure A6A–E).

#### 3.2.4. Airway Permeability and Integrity

**Figure A7:**
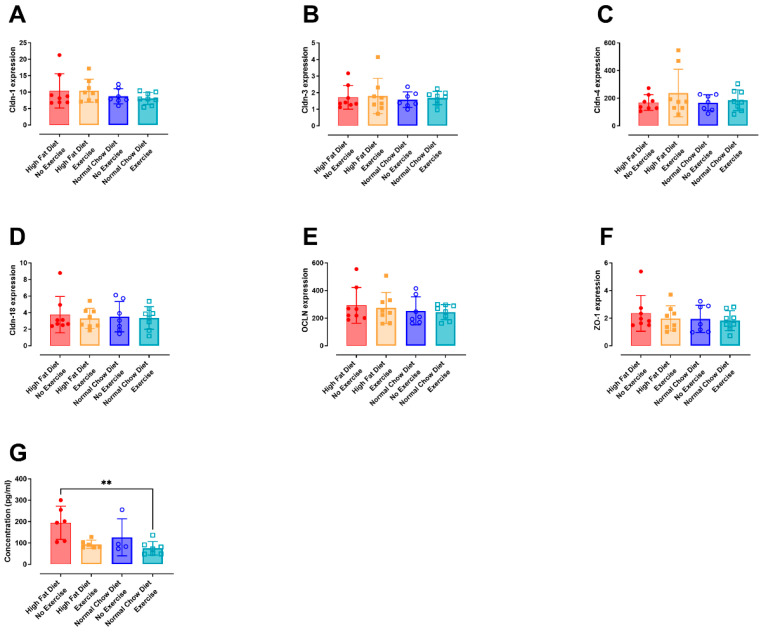
Airway permeability and integrity. mRNA expression of Cldn-1 (**A**), Cldn-3 (**B**), Cldn-4 (**C**), Cldn-18 (**D**), OCLN (**E**), and ZO-1 (**F**) in lung tissue measured with RT-qPCR. Surfactant protein D concentration in serum (**G**) was measured using ELISA (R&D systems). Data are represented as the mean ± SD with individual values. Data were evaluated using one-way ANOVA with a Dunn’s multiple comparison post hoc test. The levels of significance were ** *p* < 0.01. n = 4–8 per group.

Epithelial integrity was evaluated by measuring mRNA expression of the tight junction markers Cldn-1, Cldn-3, Cldn-4, Cldn-18, OCLN, and ZO-1 in lung tissue (Figure A7A–F). No differences between groups in any of the tight junction markers was observed.

To further investigate epithelial permeability and integrity, the leakage of surfactant protein D (SpD) from BAL to serum was assessed (Figure A7G). There was significantly more SpD leakage to serum in the HFD/NE group compared to NCD/E (*p* = 0.0068).

#### 3.2.5. Metabolic Response and Global DNA Methylation

**Figure 5:**
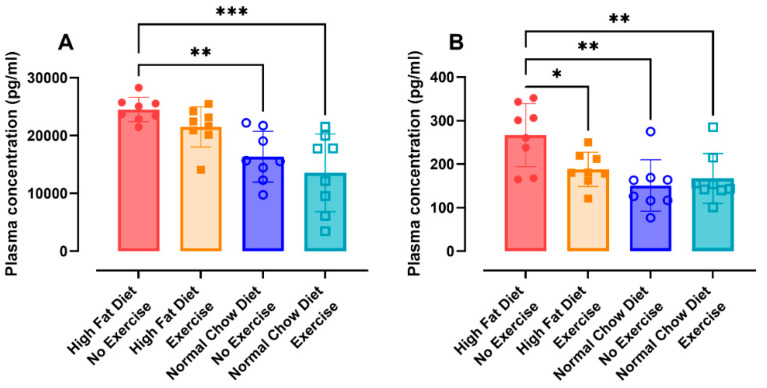
Metabolic response. The concentrations of insulin (**A**) and PYY (**B**) were measured in plasma using a U-plex Assay (Meso Scale Diagnostics). Data are represented as the mean ± SD with individual values. Data were evaluated using one-way ANOVA with a Bonferroni multiple comparison post hoc test. The levels of significance were * *p* < 0.05, ** *p* < 0.01, *** *p* < 0.001. n = 8 per group.

**Figure A8:**
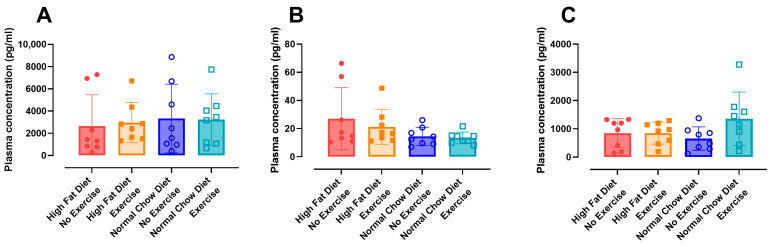
Metabolic response. Concentration of insulin (**A**), glucagon (**B**), and ghrelin (**C**) was measured in plasma using a U-plex Assay (Meso Scale Diagnostics). Data are represented as the mean ± SD with individual values. Data were evaluated using the Kruskal–Wallis test with a Bonferroni multiple comparison post hoc test. n = 8 per group.

The metabolic response towards fat restriction and exercise was evaluated in plasma. The mice that received NCD at the beginning of the treatment had a significantly lower plasma leptin concentration, independent of exercise (with exercise, *p* = 0.0001; without exercise, *p* = 0.0031) (Figure 5A). The PYY concentration in plasma was reduced in every group compared to the HFD/NE group (HFD/E, *p* = 0.0304; NCD/NE, *p* = 0.0013; NCD/E, *p* = 0.0053) (Figure 5B). No differences were found in insulin, glucagon, and ghrelin concentration (Figure A8A–C).

**Figure A9:**
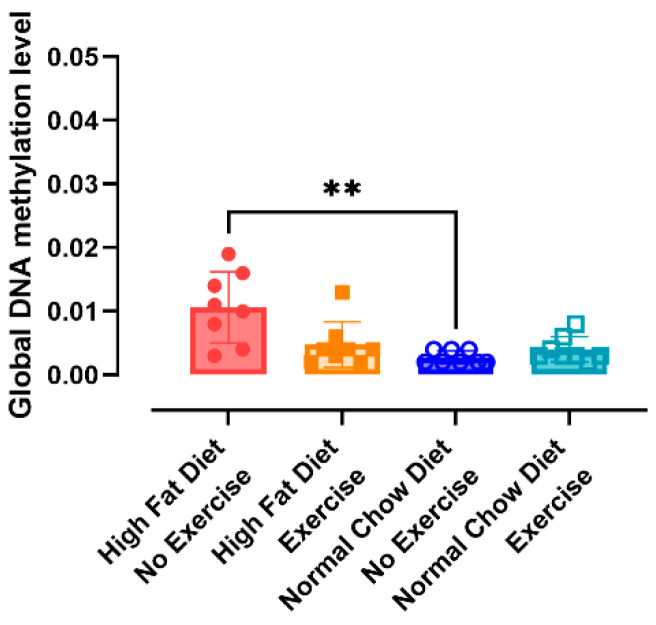
Methylation level of EDTA-coated blood pellet. 5-methylcytosine content of global DNA in EDTA-coated blood pellet was measured using MethylFlashTM Global DNA Methylation (5-mC) Elisa Easy Kit (Epigentek Group Inc.). Data are represented as the mean ± SD with individual values. Data were evaluated using the Kruskal–Wallis test with a Bonferroni multiple comparison post hoc test. The levels of significance were ** *p* < 0.01. n = 8 per group.

The 5-methylcytosine content of global DNA in EDTA-coated blood pellets was determined. The DNA methylation level was only significantly affected by diet compared to HFD/NE (Figure A9).

#### 3.2.6. Histology

**Figure 6:**
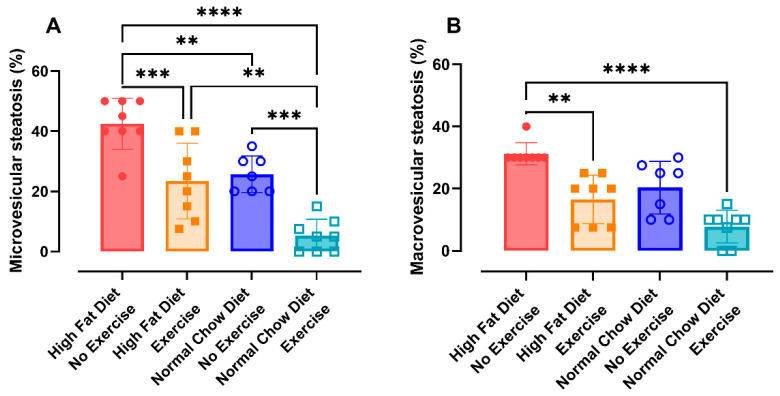
Characteristics of liver tissue. Presence of micro-vesicular steatosis (%) (**A**) and macro-vesicular steatosis (%) (**B**). Data are represented as the mean ± SD with individual values. Data were evaluated using one-way ANOVA with a Bonferroni multiple comparison post hoc test for (**A**) and using the Kruskal–Wallis test with a Dunn’s multiple comparison post hoc for (**B**). The levels of significance were ** *p* < 0.01, *** *p* < 0.001, **** *p* < 0.0001. n = 8 per group.

**Figure A10:**
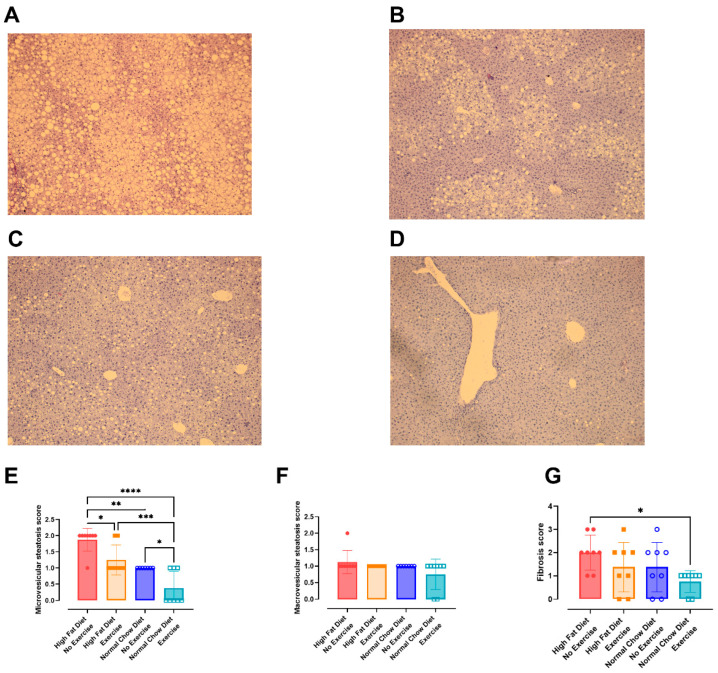
Characteristics of liver tissue. H&E-stained sections of liver tissue from HFD/NE (**A**), HFD/E (**B**), NCD/NE (**C**), and NCD/E (**D**). Magnification 10×. Presence of micro-vesicular steatosis, represented as score (**E**). Presence of macro-vesicular steatosis, represented as score (**F**). Presence of fibrosis in the liver, represented as fibrosis score (**G**). Data represented as the mean ± SD with individual values. Data were evaluated using one-way ANOVA. The levels of significance were * *p* < 0.05, ** *p* < 0.01, *** *p* < 0.001, **** *p* < 0.0001. n = 8 per group.

Histological evaluation of the liver tissue confirmed the presence of hepatic steatosis in mice on a HFD (Figure A10A). This steatosis translated into micro- and macro-vesicular steatosis. The percentage of both micro-vesicular (Figure 6A) and macro-vesicular steatosis (Figure 6B) in the liver was significantly lower after exercise alone (HFD/E) (Figure A10B, *p* = 0.0009 and *p* = 0.0072, respectively) and after combining both interventions (NCD/E) (Figure A10D, *p* < 0.0001 and *p* < 0.0001, respectively). Change from a HFD to a NCD (NCD/NE) only significantly reduced the percentage of micro-vesicular steatosis (Figure A10C, *p* = 0.0054).

When translated into a steatosis score, no differences were seen in micro-vesicular steatosis (Figure A10E). The macro-vesicular steatosis score (Figure A10F) showed similar differences as the percentual measures.

Finally, histological evaluation of the liver tissue showed that the high fibrosis score in HFD/NE was significantly reduced by combining fat restriction and exercise (NCD/E, Figure A10G) (*p* = 0.0366).

## 4. Discussion

Obesity is associated with a variety of comorbidities across different organ systems, including the liver (NAFLD) and the lungs (asthma). Recently, it was suggested that the chronic inflammatory state caused by NAFLD maintains oxidative stress and inflammation of the respiratory system [26]. However, the exact mechanisms linking hepatic injury with respiratory diseases are not understood yet. Furthermore, the effects of treating obesity on the potential resolution of comorbidities and how this impacts the association between the organ systems is not yet known.

Current non-pharmacological treatment of obesity relies on the combination of physical activity and caloric restriction [9]. Different meta-analyses evaluating human studies showed that exercise alone does not result in significant weight loss and is therefore not an effective method to treat obesity [27,28]. Furthermore, several research groups have already investigated the effect of exercise [6,21] and fat restriction [21,29] separately on different comorbidities of obesity in mice. Further research is needed to gain a better understanding on the impact of the combined treatment of physical exercise and diet. Therefore, we aimed to evaluate the effect of sub-maximal exercise with and without fat restriction on multiple organ systems involved in obesity, including the respiratory system and the liver. The main findings of this study can be found in Table A2.

Our data confirms that exercise alone was not sufficient to induce significant weight loss. Restricting fat intake by changing from a high-fat diet (HFD) to a normal chow diet (NCD) is more effective to induce weight loss than exercise alone, while combining a change of diet with increased physical activity proved the most beneficial to lose weight. For clarity, all animals kept free access to their assigned diet. Still, exercise alone, when continuously subjected to HFD, induced an average weight difference of −4.7% after three weeks of exercise, whereas animals without exercise gained on average 0.8%. This suggests that sub-maximal exercise alone can already induce clinically relevant yet not significant differences in weight without limiting access to food and fat content.

Non-alcoholic fatty liver disease (NAFLD), especially steatosis, is commonly associated with obesity. Different research groups have already established that feeding mice a high-fat diet can lead to NAFLD and non-alcoholic steatohepatitis [30,31]. Asgharpour et al. showed that animals develop steatosis from 4 to 8 weeks of treatment onwards and fibrosis from 16 weeks onwards [31]. Here, we show that changing the diet and/or implementing exercise drastically reduces the percentage of steatosis in the liver of mice. Moreover, liver fibrosis was lowered by treating obesity with combined fat restriction and exercise, shown as a significant reduction in fibrosis score. Previously, Ganguly et al. showed that non-alcoholic steatohepatitis (NASH) and fibrosis was drastically reduced after a dietary switch to NCD in Foz/Foz mice. Yet, in their model, WT mice only had very little NASH and fibrosis [32].

In our study, we found that sub-maximal exercise combined with a sudden change in diet leads to inflammatory stress in the lungs of mice, as indicated by the presence of increased neutrophils in the BAL fluid and increased concentrations of pro-inflammatory cytokines (IL-17F, IFNγ, and TNFα). Surprisingly, our mice with diet-induced obesity did not show local or systemic inflammation, contradicting previous findings [33,34].

TNFα is a pro-inflammatory cytokine that is often proposed as an important cytokine in obesity [35], in asthma [36], and in NAFLD [37]. Furthermore, previous research by Kim et al. showed a link between obesity and AHR via the TNFα pathway [22], suggesting an important role of TNFα in the pathogenesis of asthma and NAFLD. We only found a significantly higher concentration of TNFα in the BAL fluid of animals exposed to exercise and diet, which also exhibited AHR, thereby confirming the hypothesis of Kim et al. [22]. On the contrary, in plasma, we did not find differences in TNFα. This could be attributed to the short half-life of the pro-inflammatory cytokine in plasma of 18.2 min [38]. The increased pulmonary inflammation in NCD/E contrasts previous findings of Freitas et al. [39] in humans, where exercise in a weight-loss program attenuated inflammation. Yet, the inflammatory markers in Freitas et al. were measured in blood, where we did not find significant inflammation nor reduction. This could potentially be attributed to this short half-life. Moreover, Freitas et al. compared baseline measurements with post-training measurements within the same individual. In our experimental set-up, we can only compare weight to baseline measurements.

We did find a significantly higher concentration of the pro-inflammatory IL-33 in the HFD/NE group, confirming the previous findings of Gao et al. showing that HFD induces increased IL-33 in serum in mice [40]. Furthermore, Cayrol et al. previously showed an increased IL-33 concentration in animals that exhibited higher epithelial injury [41]. Likewise, we saw an increase in IL-33 concentration in the BAL of HFD/NE, which also showed increased serum SpD concentrations. SpD is produced in the airways by club cells and can leak to the vasculature when epithelial injury is present, suggesting more epithelial injury in HFD/NE [42].

The pro-inflammatory cytokine IL-4 is upregulated in the NCD/NE group. Prokopchuk et al. suggested that IL-4 can be involved in muscular adaptations [43], implying that regular training induces an anti-inflammatory environment through IL-4.

Admittedly, the analyses assessing markers related to inflammation are insufficient to make strong statements about the inflammatory profile after exercise with and without diet. Further in-depth analyses such as immunohistochemistry and mRNA expression are necessary to confirm these results. Moreover, the lack of an age-matched non-obese control group makes statements about the effects of the HFD difficult.

One of the aims of this study was to evaluate lung function and airway hyperreactivity (AHR) in diet-induced obesity. At baseline, we saw modest effects of exercise and/or fat restriction, including a lower small airway resistance after fat restriction. In contrast to other research [21], we observed limited responses to methacholine in mice with diet-induced obesity. In our model, treating obesity with sub-maximal exercise and diet induced AHR instead of reducing it. This was linked with the significant decrease in pDCs, which have been shown to have a suppressive function in asthma [44]. A possible explanation for this contradictory finding is that the intense physical exercise induced an increased concentration of pro-inflammatory cytokines, such as TNFα, and therefore resulted in AHR, while Aquino-Junior et al. showed a reduced AHR and pulmonary inflammation when diet-induced obese mice had to perform less intensive aerobic exercise [6]. In this study, animals were subjected to a running protocol whereby they had to run at 60% of their maximal running capacity for 60 min per day, 5 days a week for 5 weeks. The contradictory findings between Aquino-Junior and our study suggest that the intensity of the exercise protocol plays a major role in the effects on the respiratory system. In addition, this points out the importance of exercise stress testing. Similarly, Cho et al. [45] showed a reduction in pulmonary inflammation at the mRNA level after (moderate) aerobic exercise in mice with obesity induced by a high-carbohydrate high-fat diet. Potentially, the additional factor of changing the diet abruptly in combination with a sub-maximal exercise protocol induces a metabolic imbalance causing pulmonary inflammation in our experimental model.

Finally, Pandey et al. [46] showed that β-endorphins are able to inhibit inflammation in a murine asthma model. It is widely accepted that circulatory β-endorphins in the bloodstream are increased after exercise [47]. Therefore, there could be a protective effect to airway hyperreactivity and inflammation. Alternately, diet and caloric restrictions can reduce β-endorphin levels [48], counteracting the effect of exercise. Yet, further research is needed to confirm the effects endorphins have in this experimental set-up.

Previous research has suggested a beneficial effect of aerobic exercise on the improvement of metabolic profiles, NAFLD [49], and AHR [6]. These findings raise the question of whether sub-maximal exercise is appropriate to treat diet-induced obesity. Admittedly, the control diet used in this experiment is not ideal, since the NCD is a high-fiber diet with agricultural byproducts and therefore differs in more than only fat content [50]. Nevertheless, Almeida-Suhett et al. showed that the different control diets have similar effects on phenotypic, metabolic, and behavioral outcomes [51].

## 5. Conclusions

In conclusion, we evaluated the effect of sub-maximal exercise with and without fat restriction on different organ systems involved in obesity. We found that combining sub-maximal exercise and diet improves body weight, small airway resistance, metabolic profile, and hepatic steatosis and fibrosis, whereas it also induces modest but significant airway hyperreactivity and pulmonary inflammation. These findings suggest that combining diet and exercise is the most effective treatment to reduce obesity-associated comorbidities, such as NAFLD, while exercise intensity should be controlled. How to combine these efforts without increasing airway hyperreactivity remains to be investigated.

## Figures and Tables

**Figure A11:**
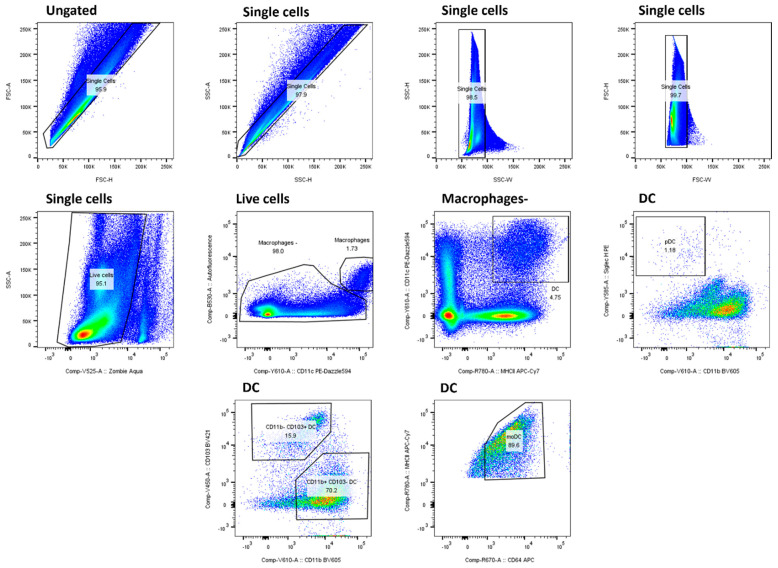
Flow cytometry data and gating for dendritic cell subpopulation in the lung. Dendritic cells were marked as CD45+ low auto-fluorescent and CD11c+ and MHCII+. Macrophages were marked as CD45+ high auto-fluorescent and CD11c+. Plasmacytoid dendritic cells (pDC) were gated as CD11b- and Siglec H+. Conventional DCs (cDC) were divided into CD11b-CD103+ cells (cDC1) and CD11b+CD103- (cDC2). Monocyte-derived DCs (moDC) were marked as CD11b+CD103-CD64+. Gating was performed based on the Fluorescence Minus One (FMO) control. Colors represent cell density in the density plot whereby blue represents low density and red represents high density.

## Data Availability

The original contributions presented in the study are included in the article, further inquiries can be directed to the corresponding author.

## Appendix A

1. Animals

During housing, animals were monitored daily for health status. Weight was measured before every running or resting session. No adverse events were observed. In advance, humane endpoints were defined as a reduction in weight of more than 20% or any severe adverse events (wounds, infection). Data were only excluded when technical errors occurred (e.g., flexiVent malfunction, poor quality of bronchoalveolar lavage (BAL) or blood sample, inability to score histological samples, measurements outside detection limit, insufficient RNA quality). For lung function measurements, one animal in the NCD/E group was completely excluded due to baseline technical errors of the flexiVent. For the dose–response curve, one additional animal in NCD/E and one animal in NCD/NE were excluded due to a buildup of fluid in the tubing. In all groups, several animals were excluded in surfactant protein D analysis due to concentrations outside the detection limit (2 in HFD/NE, 2 in HFD/E, 4 in NCD/NE, and 1 in NCD/E). One sample in NCD/NE had insufficient RNA quality to use in qPCR analyses. Lung function measurements were performed blinded by an operator unaware of the intervention method and in random order to eliminate the effects of the diurnal cycle. BAL samples were counted blinded before the treatment group was revealed.

Sample size calculations were performed based on the development of airway hyperreactivity (AHR) in previously performed experiments in our lab. AHR (indicated as area under the curve of airway resistance) was used for the sample size calculation: using an unpaired *t*-test, with a power of 80%, an error of 0.05, an effect size of 3, and a standard deviation of 2 resulted in a sample size of 7 animals per group. Considering a dropout ratio of 10% based on technical issues, we estimate that 8 animals per group is sufficient.

2. Dendritic cell subpopulations

A total of 1 million cells of the lung suspension were incubated with an anti-CD16/CD32 antibody (Clone 2.4G2, BD Biosciences, Erembodegem, Belgium) to block nonspecific binding. Subsequently, the Zombie AquaTM Fixable Viability Kit was used to stain for viability. Next, a combination of anti-mouse fluorochrome-conjugated monoclonal antibodies, as previously described [22] was used to identify DC subpopulations. Samples were analyzed on a LSR Fortessa SORP flow cytometer running FACSDIVA software (version 8.0, BD Biosciences, Belgium). Data analysis was performed with FlowJo software (version 10.10.0, BD Bioscience, Ashland, Oregon, USA). The gating strategy is available in Figure A11.

## Appendix B

**Table A1:** Overview of detection limits of different markers measured with MSD.

Marker	Detection Limit (pg/mL)	Measured In
IFN-γ	0.206	BAL supernatant
IL-13	6.26	BAL supernatant
IL-17A	0.0785	BAL supernatant
IL4	0.289	BAL supernatant
IL-5	0.225	BAL supernatant
IL-6	3.53	BAL supernatant
IL-33	0.184	BAL supernatant
IL-17F	32.6	BAL supernatant
KC	0.152	BAL supernatant
TNF-α	0.280	BAL supernatant
IL-1β	0.494	Plasma
IL-6	11.8	Plasma
IL-17A	0.634	Plasma
IL-17F	1.17	Plasma
TNF-α	1.28	Plasma
Ghrelin	23.9	Plasma
Glucagon	0.120	Plasma
Insulin	14.9	Plasma
Leptin	28.0	Plasma
PYY	4.42	Plasma

**Table A2:** Main findings of the study.

	Exercise Effect	Diet Effect	Combined Effect
Lung function			
Inspiratory capacity	↑	-	-
Tissue elasticity	-	-	↓
Airway hyperreactivity	-	-	↑
Small airway resistance	-	↓	-
Airway inflammatory response			
BAL neutrophils	-	-	↑
Inflammatory cytokines	-	-	↑
Airway permeability and integrity			
Tight junction expression	-	-	-
SpD ratio	-	-	-
Metabolic response			
Leptin	-	↓	↓
PYY	↓	↓	↓
Liver function			
Micro-vesicular steatosis	↓	↓	↓
Macro-vesicular steatosis	↓	↓	↓

Table A2 shows the major changes induced by exercise and diet separately and when combined. ↑ shows a significant increase in comparison to HFD/NE, while ↓ shows a significant reduction compared to HFD/NE. - means no differences were seen between groups.
